# The Secretive Life of Neutrophils Revealed by Intravital Microscopy

**DOI:** 10.3389/fcell.2020.603230

**Published:** 2020-11-10

**Authors:** Katia De Filippo, Sara M. Rankin

**Affiliations:** Faculty of Medicine, National Heart and Lung Institute, Imperial College London, London, United Kingdom

**Keywords:** intravital microscopy, neutrophil subsets/heterogeneity, neutrophil mobilization, neutrophil migration, tissue resident neutrophils

## Abstract

Neutrophils are the most abundant circulating leukocyte within the blood stream and for many years the dogma has been that these cells migrate rapidly into tissues in response to injury or infection, forming the first line of host defense. While it has previously been documented that neutrophils marginate within the vascular beds of the lung and liver and are present in large numbers within the parenchyma of tissues, such as spleen, lymph nodes, and bone marrow (BM), the function of these tissue resident neutrophils under homeostasis, in response to pathogen invasion or injury has only recently been explored, revealing the unexpected role of these cells as immunoregulators or immune helpers and also unraveling their heterogeneity and plasticity. Neutrophils are highly motile cells and the use of intravital microscopy (IVM) to image cells within their environment with little manipulation has dramatically increased our understanding of the function, migratory behavior, and interaction of these short-lived cells with other innate and adaptive immune cells. Contrary to previous dogma, these studies have shown that marginated and tissue resident neutrophils are the first responders to pathogens and injury, critical in limiting the spread of infection and contributing to the orchestration of the subsequent immune response. The interplay of neutrophils, with other neutrophils, leukocytes, and stroma cells can also modulate and tune their early and late response in order to eradicate pathogens, minimize tissue damage, and, in certain circumstances, contribute to tissue repair. In this review, we will follow the extraordinary journey of neutrophils from their origin in the BM to their death, exploring their role as tissue resident cells in the lung, spleen, lymph nodes, and skin and outlining the importance of neutrophil subsets, their functions under homeostasis, and in response to infection. Finally, we will comment on how understanding these processes in greater detail at a molecular level can lead to development of new therapeutics.

## Introduction

Neutrophils are the most abundant circulating leukocyte within the blood stream and play a critical role as part of the innate immune system as the first responders to infection. Clinically a blood neutrophilia is recognized as a sign of infection and the presence of large numbers of these cells in tissues observed in histology slides is a sign of infection or an on-going chronic inflammatory disease, e.g., acute respiratory distress syndrome (ARDS), chronic obstructive pulmonary disease (COPD), and the most severe cases of asthma (Kamath et al., 2005; Hughes et al., 2019; Potey et al., 2019). While neutrophils are essential for the resolution of infections their presence and activation in tissues in the context of inflammatory diseases, leads to tissue damage, thus identifying the molecular mechanisms regulating their trafficking and activation is central to the development of drugs that can limit their recruitment and activation, for the treatment of these inflammatory diseases.

The introduction of intravital microscopy (IVM) has changed our static view of approaching the study of the immune system, allowed the study of neutrophil trafficking in real time, and led to seminal work identifying the role of specific adhesion molecules, chemokines, cytokines, and signaling molecules in orchestrating neutrophil rolling, adhesion, and migration across postcapillary venules to accumulate in tissues.

The early IVM experiments were performed using fluorescent cellular dyes such as rhodamine 6G and acridine orange that labeled nuclei or intracellular organelles in all leukocytes (Mempel et al., 2004; Taqueti and Jaffer, 2013). The not selectivity of specific leukocyte subsets and the high excitation required to detect fluorescent signal caused photodamage and altered leukocyte adhesion and microvascular fluidity (Saetzler et al., 1997). Later studies used genetically modified mice that were generated as tools for *in vivo* imaging of neutrophil behavior (Stackowicz et al., 2019). In the Lys-EGFP^+^ mice, both neutrophils and monocyte are fluorescently labeled, neutrophils exhibiting high fluorescence, while monocytes are only dim green (Faust et al., 2000). However, *Listeria* inflammation has revealed a caveat of this mouse model because during infection, both neutrophils and monocyte upregulate expression level of EGFP becoming indistinguishable (Waite et al., 2011). The mouse model called Catchup overcomes this problem because only neutrophils are fluorescently labeled in red (Hasenberg et al., 2015). Fluorochrome-conjugated Ly6G mAbs are also highly used. It has been shown that low doses of i.v. injected Ly6G mAb labels neutrophils for several hours without affecting their migratory behavior and recruitment during inflammation (Yipp and Kubes, 2013).

Intravital microscopy allows imaging at microscopic resolution of ∼1 μm and temporal resolution of millisecond within the same animal allowing live cell tracking for several hours and longitudinal sessions are also possible (Masedunskas et al., 2012). The tissue penetration depth intrinsically depends on the optical properties of the tissue imaged, with transparent tissues highly penetrable, up to 300–500 μm, while highly vascularized tissue and tissue with air/liquid interface among the less penetrable (Choo et al., 2020). Limited penetration depth together with a small field of view (FOV) to image live cell behaviors are the limitations of IVM. Among the advantages, the real-time monitoring for several hours allows the design of the experiment to include homeostasis imaging followed by tissue insults or injection of pathogens in vessel or parenchyma allowing for internal controls, increasing reproducibility and importantly reducing the number of animals used. IVM allows for the long-term observation of neutrophils within different tissues of living animals giving the chance to study their behavior in the context of different physiological environments and also during different stages of diseases. Imaging of multiple channels allows for several features such as different cell types, tissue structures, or adhesion molecules to be achieved simultaneously through systemic injection of fluorescent dyes, specific mAbs, or making use of genetically modified mice in which fluorescent tags have been inserted in cell-type specific genes. Microscopes equipped to acquire several frames/s are essential to capture the single cell dynamics of highly migrating neutrophils, the interaction of neutrophils with other cells of the immune system and stromal cells and to generate quantitative data with respect to neutrophil numbers, velocity, and migratory behavior. The versatility of applying live imaging to almost all organs has also unraveled that neutrophils have tissue-specific functions and migratory behaviors increasing our knowledge on the first line of tissue protection. Further with current interest in neutrophil heterogeneity IVM allows characterization of the response of neutrophil subtypes and has provided much of the emerging evidence that indicates that the local milieu of a tissue constitutes a microenvironment capable of influencing and modulating immune cell functions. Finally, in addition to the tissue environment and neutrophil subtype being studied, there is now a convincing body of work that shows that neutrophil dynamics in health and disease are governed by circadian rhythms.

## Neutrophils Origin and Mobilization From the BM

Hematopoietic stem cells (HSCs) that exhibit a low proliferative activity but have a high self-renewal capacity are present in stem cell niches in the bone marrow (BM) and give rise to neutrophils by the process of haematopoiesis. Thus in the BM all the different stages of neutrophil differentiation are present; the multipotent high proliferative/lower self-renewal granulocyte-monocyte progenitors (GMP), granulocyte-committed progenitor myeloblasts, neutrophil-committed promyelocyte, myelocyte and metamyelocyte, finally differentiating from the immature so-called band-form neutrophils to the fully mature segmented neutrophils, these names referencing the shape of the nucleus (Borregaard, 2010; Tak et al., 2013). A recent study, making use of Fucci-(S-G2-M) reporter mouse, in which immune cells undergoing the S, G2, and M phase of the cell-cycle are fluorescent, has allowed identification, in the BM of the last three steps of neutrophil maturation process (after GMP stage), named preNeu, immature Neu, and mature Neu with unique surface marker signatures and proliferative capabilities (Evrard et al., 2018). PreNeu was identified as a proliferative committed neutrophil precursor, Fucci^+^, expressed Ly6G^*low*^/CXCR2^–^/c-Kit^+^/CXCR4^+^. Among the non-proliferative neutrophils, Fucci^–^, were immature Neu (Ly6G^*low/*+^/CXCR2^–^/CD101^–^) and mature Neu (Ly6G^+^/CXCR2^+^/CD101^+^), the latter genetically similar yet not identical to the circulating neutrophils (Evrard et al., 2018). These three stages of neutrophil maturation were also found in human BM. Xie et al. (2020) have profiled more than 25,000 mouse neutrophils using single-cell RNA sequencing and found four different clusters of neutrophils present in the BM, called G1-4, partially overlapping with the ones identified by Evrard et al. (2018). They constitute four different sequential stages in the process of neutrophil maturation and up to 24 genes were differentially expressed in each subpopulation. G1 is the more proliferative and less differentiated cluster while G4 showed the higher maturation profile constituting the fully mature neutrophil subpopulation in the BM (Xie et al., 2020).

**FIGURE 1:**
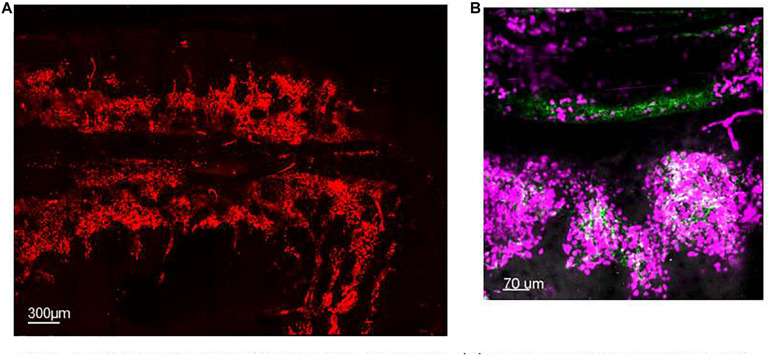
Spatial organization of neutrophils in the BM. **(A)** Maximum projection of IVM tile scan image showing the spatial organization of neutrophils within the calvarium of mice (red). **(B)** Maximum projection of IVM tile scan image of the calvarium of mice showing clusters of neutrophils (magenta) tightly organized around BM blood vessels (green).

Not all mature neutrophils egress from the BM into the circulation immediately upon maturation. A significant number of mature neutrophils are retained within the BM, referred to as the BM reserve, indeed in mice the size of the reserve is such that the ratio of mature neutrophils between the BM and blood is 300 to 1 (Furze and Rankin, 2008a). CXCL12 is a chemokine generated constitutively in the BM and evidence suggests that expression of its receptor, CXCR4, by neutrophils is critical for their retention in the BM both in human and rodents (Lapidot and Kollet, 2002; Martin et al., 2003; Eash et al., 2009) reviewed in De Filippo and Rankin (2018). Thus genetic deletion of CXCR4 was shown to result in a shift of the pool of mature neutrophils from the BM to the circulation without affecting the life-span of neutrophils (Eash et al., 2009). Static visual imaging of calvarium (Figure 1A) or long bones (Evrard et al., 2018) revealed that BM neutrophils are organized in clusters around the BM vasculature (Figure 1B) (Evrard et al., 2018) and within CXCL12-rich niches pointing to the key function of this homeostatic chemokine in neutrophil retention (Lapidot and Kollet, 2002; De Filippo and Rankin, 2018). BM-mature neutrophils are retained in large reservoirs until their need in the periphery arise to either replace dying neutrophils or to increase mature neutrophil to support the fight during infection. Despite the daily production and release of ∼10^11^ neutrophils in human and ∼10^7^ neutrophils in mice (Furze and Rankin, 2008b) to support the turnover of these short-lived cells within the whole body, applying IVM to a FOV in the calvarium or longer bones has proven that neutrophil mobilization under homeostasis is a surprisingly rare event, difficult to capture (Kohler et al., 2011; Devi et al., 2013; Pillay et al., 2020). This could be explained by the vast extension of the BM tissue that covers the cavity of the host skeleton as compared with the small FOV imaged at any one time. Thus, for example, one femur represents only 7% of the entire BM in the mouse. A similar result was obtained when studying neutrophil mobilization by transmission electron microscopy, in that while neutrophil egress could be observed it was a rare event (Burdon et al., 2008). IVM applied to the tibia BM to study the migratory behavior of single neutrophils has shown that only 30% of the entire BM neutrophils within the FOV have a basal motility of 1.5 μm/min without directionality (Kohler et al., 2011) suggesting a high level of quiescence and a minimal level of motility of neutrophils within their BM niche. During inflammation, external cues, such as granulocyte-colony-stimulating factor (G-CSF) (Semerad et al., 2002) or neutrophil chemoattractants such as CXCL1-2 can cause a rapid release of neutrophils from the BM (Burdon et al., 2008; Wengner et al., 2008; Kohler et al., 2011). Kohler et al. (2011) applying IVM to long bones have visually showed for the first time that a single systemic dose of G-CSF was sufficient to induce an increase in the motility of 75% of BM neutrophils increasing their velocity of migration up to 5 μm/min and consequently causing the egress of neutrophils from the BM into the blood stream.

AMD3100 (Plerixafor) is a CXCR4 antagonist that successfully corrects circulating numbers of neutrophils in neutropenic patients with WHIM syndrome that has been approved for clinical use (McDermott et al., 2014, 2019). WHIM syndrome is caused by a genetic mutation of the CXCR4 gene causing a gain-of-function and consequently an impairment of neutrophil mobilization from the BM that ultimately results in blood neutropenia (Hernandez et al., 2003; Gulino et al., 2004). There is controversy in the literature on whether AMD3100 stimulates neutrophil mobilization directly from the BM or from other tissues, like the lung Martin et al. (2003) and Liu et al. (2015) support mobilization from the BM while Devi et al. (2013) support neutrophil de-margination from the lung microvasculature. Using IVM of the mouse calvarium and lung, we have recently directly shown that a single dose of AMD3100 causes an increase in BM neutrophil motility observed within 30 min and mobilization from the BM niche without causing neutrophil de-margination from the lung (Pillay et al., 2020).

The process of mature neutrophil release from the BM under homeostasis is not constant during the day but fluctuates according to the circadian rhythm with a maximum mobilization during the night in mice guaranteeing the maximum number of “fresh” circulating neutrophils when these nocturnal animals are active (Casanova-Acebes et al., 2018). This is also the case for humans, but with the clock inverted (Adrover et al., 2020). The impact of circadian rhythms on neutrophil mobilization, clearance, and inflammation has been more extensively reviewed here (Scheiermann et al., 2013; Hidalgo et al., 2019; Adrover et al., 2020; Aroca-Crevillen et al., 2020).

With respect to the BM and neutrophil mobilization one question that still remains is whether all the mature neutrophils mobilized are identical or whether there are neutrophil subsets within the BM that can be differentially mobilized.

The BM is also one of the major organs where some senescent neutrophils home back at the end of their life span to be cleared by stromal macrophages and this process feeds back on the maturation of neutrophils (Gordy et al., 2011). We will discuss this in more detail later in the senescent session of this review.

## Circulating and Marginated Pools of Neutrophils

Electron microscopy has shown that neutrophil extravasation from the BM occurs through specialized endothelial cells that exhibit diaphragmed fenestra. Neutrophils transmigrate across this BM sinusoidal endothelium through small pores in a transcellular manner (Burdon et al., 2008). This marks the “birth” of neutrophils passing from their niche in the BM into the blood stream where they are exposed to sheer forces. Mature neutrophils kept in the BM are only genetically similar yet not equal to circulating neutrophils (Evrard et al., 2018) suggesting that the environment may impact on neutrophils and induce their genetic changes. This concept is also indirectly supported by the change of surface markers expressed by neutrophils when they enter circulation (Evrard et al., 2018; He et al., 2018; Adrover et al., 2020). Based on the differential expression of more than 20 genes, in peripheral blood, three different subpopulations (G5a–c) have been identified. Xie et al. (2020) showed that blood G5a and G5b are two independent subsets of neutrophils that differentiate from G3 and G4 BM clusters, respectively.

Neutrophils are the most abundant leukocyte circulating within the blood. In this state, neutrophils do not interact with other cells and flow at a fast speed passively transported by the blood stream. The circulating number of neutrophils is the result of a fine balance between neutrophils that are mobilized from the BM (input) and neutrophils that emigrate into tissues or are cleared (output). This must be tightly regulated to avoid excessive numbers of circulating neutrophils or unregulated activation of neutrophils that could lead to vascular or tissue damage. Circadian release of neutrophils from the BM is reflected in the circadian oscillation of neutrophil number in the blood with a high number of aged neutrophils during the day and a high number of fresh neutrophils during the night in mice (Casanova-Acebes et al., 2013). These oscillations of neutrophil number are conserved among species and in human follow the opposite pattern to rodents (Adrover et al., 2020). Using an organism-wide circadian screening approach, it has been shown that circulating neutrophils in different stages of their life specifically upregulate or downregulate surface markers, including specific adhesion molecules and chemokine receptor CXCR4, that dictate their vasculature or tissue infiltration in a time-of-day-dependent manner during homeostasis and inflammation (He et al., 2018).

Within special vascular beds, under homeostasis, neutrophils can be found in direct contact with ECs, this population of neutrophils is referred to as the “marginated” or “intravascular retained” pool. The major organs where neutrophil localize within the microvessels of the blood are the lung (Gee and Albertine, 1993; Gebb et al., 1995) and the liver (Casanova-Acebes et al., 2018). It has been shown that circulating and intravascular retained neutrophils are at equilibrium and that adrenaline and physical exercise can increase the circulating pool of neutrophils, by releasing neutrophils from this intravascular compartment (Summers et al., 2010). Whether intravascular retention is an active process mediated by adhesion molecules or a passive process due to the mechanical constriction of these cells as they move through small microvessels is still under debate. However, several studies, making use of genetically modified mice, have shown that L-selectin is not essential for neutrophil margination (review Doerschuk, 2001; Kolaczkowska and Kubes, 2013). Likewise, direct imaging of lungs by IVM showed that CD11b expression on neutrophils and the β_2_ integrin are not required for neutrophil margination in the microvessels of the lung but play an essential role during neutrophil locomotion toward systemic pathogens captured by lung ECs (Yipp et al., 2017).

ECs differ between tissues and have been shown to utilize different adhesion molecules to sustain neutrophil adhesion (Kolaczkowska and Kubes, 2013). Likewise, neutrophils themselves are plastic and highly adaptable cells equipped with different surface molecules so that they can adapt to interact with different types of ECs.

## Tissue Neutrophil Dynamics Under Homeostasis

The dogma that under homeostasis tissues were virtually free of neutrophils was challenged by parabiotic experiments and IVM. Both these techniques directly showed that neutrophils infiltrate almost every naïve tissue apart from the reproductive organs and the brain (Casanova-Acebes et al., 2018). Moreover, the level of neutrophil infiltration is tissue-specific, with BM, spleen, lung, and liver among the highest neutrophil infiltrated organs, suggesting that these tissue resident neutrophils may support tissue regulatory functions under homeostasis, and/or provide immune protection and surveillance under steady state (Casanova-Acebes et al., 2018). The spleen and intestine harbor neutrophils within the tissue parenchyma as an integral part of these organs elevating their surveillance functions and roles (Puga et al., 2011; Casanova-Acebes et al., 2013). As with the rhythmic fluctuation of circulating neutrophils, the absolute number of neutrophils retained in these tissues oscillates with a maximum retention within the tissues during the dark phase mirroring their maximum numbers in the blood (He et al., 2018). The circadian changes in neutrophil retention in tissues were shown to be under the control of adrenergic nerves, changing the expression of adhesion molecules, including ICAM-1, VCAM-1, P- and E-selectins, and MadCAM on the surface of ECs (Scheiermann et al., 2012) with a maximal expression at night in mice, promoting the release of neutrophils into the blood (He et al., 2018). Thus, in addition to fundamental differences in tissue microenvironments and the heterogeneity of ECs, changes in adhesion molecules driven by circadian rhythm all combine to determine neutrophil numbers in the blood and different tissue during homeostasis (Kolaczkowska and Kubes, 2013).

## Neutrophil Dynamics in Response to Pathogens

Neutrophils play a pivotal role as the first line of innate immune defense and constitute the first cells to be recruited within an infected tissue. In the absence of neutrophils, life will consist of recurrent and life frightening infections. Numbers of circulating and tissue-recruited neutrophils increase substantially during infection by several mechanisms: increased mobilization form the BM (Christopher and Link, 2007; Burdon et al., 2008), increased adhesion and transendothelial cell migration through ECs to tissues (Ley et al., 2007), and also by the extended neutrophil half-life (Tak et al., 2013; Silvestre-Roig et al., 2016). Neutrophil activation during infection has been proposed to happen via two sequential stages: priming and full activation (Summers et al., 2010; Kolaczkowska and Kubes, 2013). Neutrophils are primed by their exposure to mediators such as cytokines; increased surface expression of CD11b and decreased of L-selectin are features of activated neutrophils.

Intravital microscopy of the blood vessels in the cremaster muscle was one of the first models to be established and extensively used to study neutrophil interaction with the venular walls because of the accessibility and the transparency of the organ allowing imaging via transmitted light. This model has been used extensively to image and define the molecular mechanisms involved in the sequential phases of the leukocyte adhesion cascade, where selectins, chemokine receptors, and β_2_ integrins as well as JAMs and PECAM are sequentially activated to guarantee neutrophil capture, rolling, firm adhesion, and transendothelial migration from the circulation into the infected tissue (Ley et al., 2007; Evans et al., 2009). Within activated vessels, neutrophils organize membrane protrusions at the back of the neutrophil termed the uropod that are rich in selectin ligand PSGL-1, and shown to transiently interact with activated platelets (Sreeramkumar et al., 2014). Using IVM, the PSGL-1 rich domains on neutrophils could be observed either protruding into the lumen of the blood vessels or laterally toward the endothelium to facilitate neutrophil interaction with platelets and ECs, respectively. It is the integrated signal from both activated vascular endothelium and platelets that triggers and supports neutrophil crawling within the activated blood vessels, an essential step that precedes neutrophil extravasation (Sreeramkumar et al., 2014).

Tracking neutrophil migratory behavior showed that their extravasation during challenge happens in the post capillary venules in the systemic circulation predominantly via a paracellular route and requires ∼6 min to complete (Woodfin et al., 2011). Infected tissues generate inflammatory molecules that on one hand instruct ECs to upregulate the expression of adhesion molecules to sustain vascular attachment of neutrophils, and on the other hand cause the direct activation of neutrophils and their increased diapedesis (Ley et al., 2007; Nourshargh and Alon, 2014). Moreover, IVM applied to this model has been essential to study the efficient and persistent neutrophil transmigration dissecting the sequential and unique role of the neutrophil selective chemokines, CXCL1 and CXCL2 during neutrophil diapedesis (Girbl et al., 2018). Girbl et al. (2018) showed that CXCL1, mainly produced by ECs, supported luminal and sub-EC crawling, whereas CXCL2, mainly produced by neutrophils, was essential for self-guiding neutrophils in breaching through EC junctions.

The process of neutrophil adhesion and transmigration has also been imaged in real time in an *in vitro* system of flowing human neutrophils across stimulated EC-cultured glass capillaries (Buckley et al., 2006). Using this system, neutrophils were observed to reverse the process of transendothelial migration, in a process referred to as reverse transmigration (R-TEM). IVM imaging of the cremaster muscle showed that this was not an *in vitro* artifact but occurred *in vivo* too.

Further, R-TEM was found to be a β_1_-, β_2_-, and β_3_-integrin-independent mechanism. It was found that only neutrophils that phenotypically are ICAM-1^*high*^ CXCR1^*low*^ were able to reverse transmigrate. Tissue resident neutrophils are ICAM-1^*low*^ CXCR1^*low*^ while naïve circulating neutrophils are ICAM-1^*low*^ CXCR1^*high*^ therefore incapable of R-TEM (Buckley et al., 2006). As discussed above, high levels of the adhesion molecule JAM-C at the junctions between ECs constitute a physical barrier that regulates the polarized unidirectional transmigration of neutrophils from the lumen of vessels to the tissue (Ley et al., 2007). Loss of JAM-C by ECs at their junction was associated with a “hesitant” and R-TEM leading to the systemic dissemination of activated tissue-experienced neutrophils causing second organ injury specifically in the lung (Woodfin et al., 2011). Mechanistically it was found that LTB4 dependent release of neutrophil elastase (NE) lowered the levels of JAM-C on postcapillaries ECs, thus neutrophil activation was required to support their own R-TEM (Colom et al., 2015). Neutrophils that underwent R-TEM were uniquely characterized by a prolonged lifespan and an inflammatory phenotype. They were found in distal organs like the lung causing tissue inflammation thereby disseminating the extent of organ injury (Woodfin et al., 2011; Hossain and Kubes, 2019).

In an infection model of *Candida albicans*, the time of day-night when the infection happens had a dramatic impact on the outcome of infection. Administration of infection during the night, when the number of aged neutrophils in the tissue was at its peak, conferred resistance to Candida (Adrover et al., 2019). The same was true in an intraperitoneal model of LPS, with the peak of recruited cells from the blood to the tissue during the dark phase (He et al., 2018). Collectively these circadian studies stress the fact that experimental variability in the number of recruited neutrophils to specific tissues could be related to the time of day when experiments are performed.

It is thought that resident populations of neutrophils in tissues can be rapidly deployed to mount a localized host response to pathogens, thus marginated neutrophils in the lung microvasculature constitute a critical part of the rapid lung immune response. Very little is currently known about the turnover of tissue resident neutrophils during homeostasis and the mechanisms required to keep these highly cytotoxic cells from causing tissue damage. During *Escherichia coli* challenge, it was shown that the different neutrophils subsets were still present but the transcriptional activity of several genes increased in each population (Xie et al., 2020).

Apart from this rapid deployment of neutrophils from the periphery to the site of infection, the function and role of tissue resident neutrophils need to be determined. One obvious function is that there is a population of neutrophils already within the local environment ready to respond to insults, thus increasing the speed of the immune response of the host in the tissues. In theory an even more effective level of tissue protection will result if these tissue resident neutrophils have been previously “instructed” by tissue resident stroma cells. How the tissue instructs neutrophils during homeostasis and how the neutrophils exist within the tissue without inflicting damage during homeostasis are still not fully understood.

Is it extensively documented that during systemic acute diseases, such as endotoxemia and chronic diseases, such as asthma and cystic fibrosis (CF), neutrophils showed plasticity with the appearance in both mouse and human of neutrophil subsets, identified by differential expression of surface molecules including, CD177, CD49d, VEGF-R1, CD11b, CD18, and CXCR4, and displaying different functional responses, review by Silvestre-Roig et al. (2016). Thus airway neutrophils from asthmatics and patients with CF exhibit metabolic reprogramming and a substantially prolonged lifespan, both of which have been shown to contribute to disease severity (Garlichs et al., 2004; Uddin et al., 2010; Laval et al., 2013). Moreover, transcriptional plasticity has been reported for neutrophils during inflammation and disease, suggesting that neutrophils adapt and respond to local environmental cues (Silvestre-Roig et al., 2016). Taken together these studies demonstrate that neutrophils exhibit plasticity and heterogeneity in the context of an inflammatory tissue environment. However, as yet it is not known whether the heterogeneity is intrinsic in neutrophils at birth or acquired during tissue persistence.

Many other factors can affect neutrophil trafficking including age, gender, diet, gut microbiota, metabolism, or genetics but these are outside the scope of this review.

One limitation of IVM is the number of cell types/surface adhesion molecules that can be directly and longitudinally tracked at any one time, due to the number of channels that can be imaged simultaneously. Currently usually three to four channels are imaged simultaneously by fluorescent IVM. To overcome this limitation, there is a move to use simultaneous label-free autofluorescent-multiharmonic microscopy (SLAM). This technique relies on the fact that different cells and ECM can be identified by their morphology, autofluorescence, and harmonic generation, thus there is no need to fluorescently label cells of interest. This technique can be used to visualize cell–stroma interactions, tissue remodeling, and metabolic activity (You et al., 2018). In one study using SLAM, it was shown that while the number of leukocytes increased and formed clusters in tissues in response to LPS, the density of collagen fibers and lipids decreased leaving space for the recruited leukocytes (You et al., 2018). Moreover, SLAM revealed that the local tissue environment was hypoxic and that the clustered leukocytes showed a reduced redox ratio indicating an increase in metabolic activity (You et al., 2018). Thus SLAM provides different information to IVM; however, it lacks the ability to unequivocally identify leukocyte subtypes or different subsets of neutrophils, thus SLAM could constitute a complementary approach to use alongside IVM.

## Neutrophil Margination in the Capillary of the Skin and Migration Within the Derma-Skin After Challenge or Damage

The skin is a vast organ at the interface with the outside world and a robust level of defense must be provided to protect and quickly respond to potential harmful invading pathogens. Because vessels and tissue can be imaged directly through the skin, no surgery is required. Imaging of the mouse footpad has been extensively used as a model to unravel neutrophil migratory behavior in the derma (Zinselmeyer et al., 2008), while a more recent advanced method has been developed to study the epidermis and dermis of the mouse ear and dorsal skin (Li et al., 2012).

During homeostasis, visual imaging showed that neutrophils flow in the microvessels of the footpad at several hundred μm/s and rarely adhere to the endothelium of the skin (Zinselmeyer et al., 2008). Neutrophils reside transiently and in a small number within the connective tissue of the skin-derma and constantly exit via lymphatics during homeostasis (Ng et al., 2011) constituting a direct line of tissue protection.

The use of IVM to study the neutrophil dynamics during subcutaneous injection of parasites *Leishmaniasis* has shown that neutrophils are involved in both protection of the host and disease progression (Peters et al., 2008). Thus 30 min post infection, IVM reveals a substantial accumulation of neutrophils inside the blood vessels near the infected area and subsequent diapedesis into the skin parenchyma (Peters et al., 2008; Zinselmeyer et al., 2008; Graham et al., 2009). Using IVM the interplay of neutrophils and macrophages in the clearance of parasites from the skin was observed, showing that apoptotic neutrophils decrease their velocity from 4–14 to 0–6 μm/min and release the parasites in the vicinity of the tissue macrophages for final clearance (Peters et al., 2008). However, surprisingly, in a neutrophil-depleted model, the dissemination and survival of parasites was reduced suggesting that neutrophils can also act like a “Trojan horse” supporting the spread of the live pathogens to distant organs (Peters et al., 2008). In a similar manner, Duffy et al. (2012) observed that during a dermal viral infection, neutrophils were able to migrate in a CCR1-dependent manner from the dermis to the BM carrying the virus, again functioning like a “Trojan horse,” but in this context they interacted with antigen presenting cells (APCs) and primed BM CD8 T cells to mount an adaptive immune response. Moreover, in models of sterile dermal tissue damage caused by laser and during several cutaneous infections, single cell tracking of neutrophils by IVM revealed that they rapidly switched their probing migratory behavior during homeostasis into a highly directional mode of migration during tissue damage that reached mean velocity of 7.8 ± 2.5 μm/min (Graham et al., 2009; Ng et al., 2011), swarming around the damaged area of tissue or site of infection to clear the pathogens (Lammermann et al., 2013). By real-time investigation, Ng et al. (2011) showed that neutrophil locomotion occurs in three sequential phases with a few “scouting” neutrophils observed arriving within the first 15 min post damage, followed by an amplification phase with a synchronized attraction of a high number of neutrophils that traveled up to 150 μm and a stabilized phase in which neutrophils clustered around the damaged area. IVM allowed Lammermann et al. (2013) to show that in a radius of more than 300 μm, neutrophils sensed and directionally migrated toward the tissue lesion for up to 40 min.

In the model of *Staphylococcus aureus*, IVM was an essential tool to study the host–pathogen interaction, neutrophil migration, and abscess formation in the skin following infection (Liese et al., 2013). Moreover, IVM applied to a mouse skin flap with intact blood flow, allowed Liese et al. (2013) to dissect at the molecular level the temporal migratory dynamics of neutrophils during dermal infection showing that interstitial neutrophil migration during pathogen challenge is G-protein coupled receptor- and IL-1R-dependent process.

Intravital microscopy has also permitted the observation of neutrophil behavior within small capillaries, showing that neutrophils are dramatically elongated and are indecisively crawling back and forth over a distance of ∼100 μm closer to the subcutaneous nidus of *S. aureus* infection to prevent pathogen dissemination (Harding et al., 2014). This migratory behavior is LFA-1- and VLA-4-dependent but Mac-1-independent. Only a small number of those crawling neutrophils were observed exiting at the postcapillary venules and were recruited in the infected area (Harding et al., 2014).

## Neutrophils Marginate Within the Microcapillaries of the Lung

**FIGURE 2:**
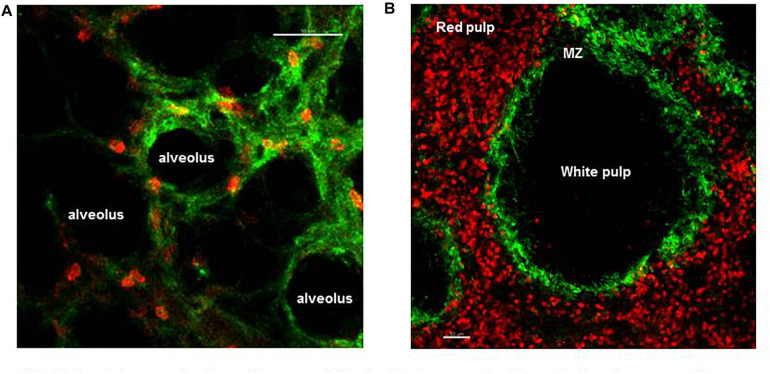
Spatial organization of neutrophils in the lung and spleen during homeostasis. **(A)** Frame from lung IVM video showing marginated neutrophils (red) within the microvessels of the lung (green). **(B)** Precision cut spleen slice showing tissue resident neutrophils (red) and margiant zone macrophages (green) delimiting the MZ. Scale bar 50 μm.

In humans, the microvessels of the lung form an intricate network of approximately 2.8 × 10^11^ capillaries covering an estimated 10^8^ alveoli (Hogg and Doerschuk, 1995), with diameters spanning from 2 to 15 μm they guaranty O_2_–CO_2_ exchange, thereby support gaseous exchange requirements for every single cell of the body. The entire output of the heart is distributed within this fine capillary network that has an extended surface area of ∼70 m^2^. As such blood velocity is dramatically reduced facilitating neutrophil–EC interaction (Gee and Albertine, 1993). Indeed, there is a large pool of marginated mature neutrophils, anchored within these microvessels under homeostasis (Figure 2A). This is important because the lung is constantly exposed to potentially harmful pathogens and pollutants/particles due to direct contact with the external environment through the air we breathe, and this marginated pool of mature neutrophils serve as a first line of defense in the lung (Gee and Albertine, 1993). Intravascular retained neutrophils are uniquely positioned within the vascular space where they remain until they are required to migrate into inflamed alveoli or parenchyma of the lung depending whether the pathogen is located in the airspace or tissue, respectively (Hogg and Doerschuk, 1995). It was 1987 when Lien et al. first made use of fluorescent video microscopy to observe labeled neutrophil migrate and interact in the sub-pleural pulmonary microcirculation through a window inserted into the chest wall of dogs. In this pioneering study, the authors observed that under homeostasis neutrophils were making transient contacts with ECs and migrated within the pulmonary capillaries with a transit time ranging from 2 s to 20 min but in contrast had minimal engagement in arterioles or venules (Lien et al., 1987). For decades the precise nature, function, and size of the intravascular marginated pool, versus the circulating pool, has divided scientific opinion; moreover, the molecular mechanism behind this interaction has not been identified (Gee and Albertine, 1993; Peters, 1998). Early studies suggested that ∼36% of cells are in circulation and ∼64% are engaged with lung ECs forming the “physiological regional pool” (Peters, 1998). The increase in IVM image resolution and stabilization of imaging allowed Looney et al. (2011) to directly image up to 125 μm below the pleura and follow in the physiologically intact lung the migratory dynamic of neutrophils up to 3 h. In this study, neutrophils were reported to traverse the 10–15 μm capillaries with a track speed of ∼1.5 μm/s, while in the medium size vessels at ∼100 μm/s confirming that neutrophils were engaging with the endothelial cells of the lung capillaries. Confocal pulmonary IVM further showed that neutrophils under homeostasis possess an array of migratory behaviors; tethering, crawling, and firm arrest but not rolling (Looney et al., 2011). Under homeostasis, these marginated neutrophils crawled a distance of only few μm (Yipp et al., 2017), but this increased significantly after intratracheal administration of LPS or *E. coli* particles reaching a mean velocity of ∼9.68 μm/min (Kreisel et al., 2010). The role of adhesion molecules in sustaining firm and prolonged interaction of neutrophils with the lung ECs has been controversial with contradicting and inconclusive results (Gee and Albertine, 1993). L-selectin deficient mice were reported to have a normal pool of intravascular retained neutrophils in the lung (Doerschuk, 2001). Assuming that the pool of marginated neutrophils consists of “non-activated” neutrophils interacting with non-activated endothelium, it does not come as a surprise that the classical molecular mechanisms associated with adhesion in the context of inflammation do not apply to this pool of neutrophils interacting with the vasculature under homeostasis. The blood flow in the microcapillaries, as discussed above, is relatively low and due to the diameter of the capillaries it is clear that neutrophils change shape to squeeze through the microcapillaries and this results in their slow transit time. It has been argued that the mechanical mechanism of cell squeezing could stimulate their margination promoting interaction with the lung capillaries, an interesting hypothesis that requires further evidence (Kuebler and Goetz, 2002; Rossaint and Zarbock, 2013). We have recently directly shown, using IVM to image the pulmonary capillary network of mice that a single dose of the CXCR4 antagonist, AMD3100, did not compromise the lung intravascular retained pool of neutrophils under homeostatic conditions (Pillay et al., 2020). Further applying dynamic planar gamma scintigraphy, we have also shown that AMD3100 does not affect the retention of primed neutrophils in the capillary circulation of the lung in humans. Taken together these data suggest that the CXCR4-CXCL12 chemokine axis does not support neutrophil retention in the lung microvascular in either mouse or human (Pillay et al., 2020). Thus to date the precise molecular mechanisms underlying the retention of mature neutrophils in the pulmonary capillaries are unknown or still remain a mystery.

While neutrophils serve as a critical line of host defense in the lung, in the context of ARDS and a number of chronic lung diseases, e.g., COPD, IPF, and asthma, it is thought that the accumulation of excessive numbers of neutrophils supports disease progression (Kamath et al., 2005; Hughes et al., 2019; Potey et al., 2019). Thus, understanding the mechanisms underlying neutrophil influx is desirable to enable the development of targeted therapeutics that can reduce neutrophil numbers in these clinical scenarios. In contrast to the situation under homeostasis, there is consistent evidence from several studies showing the requirement of specific adhesion molecules to support increased neutrophil retention within the microvessels of the lung and their further migration within the infected parenchyma (Doerschuk, 2001). The nature of the adhesion molecules is stimulus dependent, thus neutrophil emigration utilizes β_2_ integrins when elicited by *E. coli*, but not when elicited by *Streptococcus pneumoniae* (Doerschuk, 2001).

L-IVM showed that following systemic challenge, *E. coli* was sequestered within seconds by the lung ECs in the pulmonary capillary network and this was followed by the rapid migration of marginated neutrophil toward the immobilized pathogen (Yipp et al., 2017). This work indicates that the lung is an important host defense niche for the detection and capture of systemic pathogens, and requires cooperation between the vascular endothelium and marginated neutrophils (Yipp et al., 2017). In the search for new adhesion molecules that support neutrophil retention in the lung during inflammation, an *in vivo* functional screen surprisingly identified dipeptidase-1 (DEPEP1) (Rajotte and Ruoslahti, 1999; Choudhury et al., 2019). DEPEP1, a membrane enzyme expressed by activated pulmonary endothelium, was shown to support neutrophil adhesion, independent of its enzymatic activity. Moreover, genetic deletion studies and use of a blocking peptide showed that neutrophil adhesion and recruitment in the inflamed lung was significantly attenuated in the absence of DEPEP1 during sepsis (Choudhury et al., 2019). Finally, when the DEPEP1 blocking peptide was used therapeutically in mice administered with a lethal dose of LPS, it showed a remarkable survival effect and an impressive reduction in neutrophil recruitment into the inflamed lung (Choudhury et al., 2019). With respect to the focus of this review, highlighting how IVM has been key to advancing our understanding and identifying the molecular pathways regulating neutrophil trafficking, this research is notable in that the initial screen involved using confocal IVM to identify a peptide that localized to both the lung and liver endothelium after LPS treatment and reduced neutrophil accumulation in these tissues. Importantly, while this research was carried out in mice, recombinant human DPEP1 supported the adhesion of human neutrophils *in vitro*, indicating its translational potential.

While neutrophils are important for host defense, as mentioned above, when they accumulate in large numbers in tissues, they also have the potential to cause considerable damage to the host tissue. This is due to the fact that their granule proteins and neutrophil extracellular traps (NETs), both important for their anti-microbial functions, are also cytotoxic. In this respect, a series of recent discoveries, also made using IVM, provide an explanation for why marginated neutrophils in the lung may not be as cytotoxic as those in the circulation. Following their release from the BM neutrophils only circulate for 6–10 h before they exit into tissues, including the spleen, BM, and lung (Casanova-Acebes et al., 2018). The migration into the lung is regulated by CXCL1 and the clock gene BMAM-1(Adrover et al., 2020), which in the mouse, means that the majority of circulating neutrophils infiltrate the lungs during the daylight (Adrover et al., 2019). At this time, as compared to neutrophils freshly mobilized from the BM, circulating neutrophils exhibited changes in their proteome, with a reduction in cytotoxic granule proteins, which in turn reduced their ability to form NETs. This process is termed neutrophil “disarming.” Taken together, these findings explain how neutrophils can exist in large numbers as marginated cells in the microvasculature of the lungs without causing tissue damage. They also explain why acute lung injury caused by the influx of neutrophils in response to an inflammatory stimulus varies considerably dependent on the time of day. Thus in mice, LPS challenge of the lungs at night will result in greater host tissue damage, due to accumulation of neutrophils from the circulation that have a high content of cytotoxic proteins in their granules as compared to those that would accumulate during the day that have an aged phenotype with lower cytotoxic potential (Adrover et al., 2020). While these studies have been performed in mice, similar changes in neutrophil proteome have been reported human neutrophils with aging, suggesting that these findings are translatable (Adrover et al., 2020).

Another fascinating function of neutrophils has been identified by Wang et al. (2017) in their ability to promote tissue repair in a model of murine sterile thermal hepatic injury. IVM showed that following laser injury of the liver, tissue neutrophils were involved both in dismantling the injured vessels and then in directly contributing to the deposition of collagen in a honeycomb pattern to create a path to rebuild the new vasculature (Wang et al., 2017). In response to the hepatic injury, some neutrophils migrated away from the site of injury while a small number were observed to re-enter the circulation. These neutrophils were later found to “sojourn” in the lung where they upregulated CXCR4 before homing back to the BM in a final voyage to be cleared (Wang et al., 2017). While it is fascinating that a subset of neutrophils, having experienced tissue injury in one organ, makes a pit-stop in the lungs before being cleared in the BM, the reason for this process is still not fully understood. More models need to be tested in order to prove whether this is a specific mechanism that links the liver and the lung or broadly applies to any injured organ, and whether this is only linked to sterile damage or applies also to infection.

Recently, Fluorescent *influenza virus* (color-flu) has been developed as a means of studying influenza infection in the lungs of mice by IVM. Details of the model and a database of fluorescent dyes, antibodies, and reporter mouse lines that can be used in combination with Color-flu for multicolor analysis have also been reported (Xie et al., 2020). Using this model, pulmonary permeability (by dextran leakage from the lung vessels into the alveolar space) and blood flow speed (by i.v. injection of fluorescent microbeads) have been studied following infection, in addition to studying neutrophil dynamics (Ueki et al., 2018; Xie et al., 2020). Thus they reported a reduction in pulmonary permeability and blood perfusion speed during infection, highlighting the severe pulmonary damage created by the virus to the host (Ueki et al., 2018). Neutrophil dynamics exhibited a temporal change in speed, with a high migratory speed ≥ 50 μm/s during the early time points (30 min to 1 h) following virus infection and a slow migratory behavior characteristic of the later phase (Ueki et al., 2018).

## Neutrophil Margination Within the Parenchyma of the Spleen and Lymph Nodes

As a secondary lymphoid organ, an important function of the spleen is in mounting the adaptive immune response during pathogen challenge. In addition, three distinct subsets of macrophages (metallophilic, marginal, and red pulp) present in the spleen play a critical role in filtering the blood, removing senescent red blood cells and systemic pathogens. After the BM the spleen contains the largest number of neutrophils during homeostasis, but until recently the dynamics of these tissue neutrophils was unknown (Casanova-Acebes et al., 2013). IVM showed that two distinct splenic neutrophil populations, at distinct maturation stages, populate the red pulp of this organ under homeostasis (Figure 2B) and differentially respond to pathogen challenge (Deniset et al., 2017). Thus, Ly6G^*high*^ are mature neutrophils that scan the tissue at varied speeds from 0–2 μm/min and up to more than 10 μm/min under steady state. Their migratory speed declined 24 h after challenge increasing the dwelling time and number of firm interactions with local splenic macrophages. Ly6G^*intermediate*^ are immature and static neutrophils capable of undergoing emergency proliferation during pathogen challenge contributing to the removal of pathogen and of plucking *S. pneumoniae* from the surface of red pulp macrophages (Deniset et al., 2017).

The very same preNeu, immature Neu, and mature Neu that populate the BM (described in detail above) were also found in the spleen under homeostasis even if in a reduced number compared with the population in the BM (Evrard et al., 2018). During sepsis, preNeu numbers in both the BM and spleen expanded with a greater fold of increase in the spleen indicating that both organs contribute to emergency granulopoiesis in response to infections (Evrard et al., 2018) and represent a store of immature cell reserves. The possibility that neutrophils can complete the last stages of their maturation outside of the BM in the spleen also give rise to the possibility that tissue specific education may prime neutrophils such that they are better tailored to the immune surveillance property of the spleen for a fast and more effective response to pathogens or tissue damage. Puga et al. (2011) identified another distinct subset of neutrophils in the spleen, the splenic neutrophil B-helper cells (N_*BH*_), that can support marginal zone B cell maturation and induce their antibody secretion during pathogen challenge via the production of B cell-attracting chemokines such as CXCL12 and CXCL13. Confocal microscopy revealed that N_*BH*_ interact directly with MZ B cells via protrusion similar to DNA-containing-NET-like projections. Moreover, two genetically and phenotypically distinct subsets have been identified, called N_*BH1*_ and N_*BH2*_ (Puga et al., 2011). It is still unknown whether these two populations identified in humans are comparable to Ly6G^*high*^ and Ly6G^*intermediate*^ murine neutrophils identified by Deniset et al., as reviewed (Scapini and Cassatella, 2017).

The molecular mechanisms underlying the retention of splenic neutrophils are still under investigation. Applying IVM, Pillay et al. (2020) ruled out the CXCL12-CXCR4 chemokine axis-as molecular mechanism responsible of the splenic retention of neutrophil. In fact, treatment with the CXCR4 antagonist, AMD3100 caused an increase in the number of circulating and splenic neutrophils (Liu et al., 2015) as early as 30 min post challenge and while these splenic neutrophils showed an increase in their migratory speed, there was no evidence that they were activated (Pillay et al., 2020). These data suggest that the spleen can also functions as a sink, lowering the number of circulating neutrophils when they reach a specific threshold. It is possible that the pooling of neutrophils in the spleen protects other more fragile organs, such as the lung from neutrophil overload and potential damage.

Intravital microscopy also revealed that neutrophils patrol unstimulated draining lymph nodes of the skin, lung, and gastrointestinal track (Lok et al., 2019) and reside within the interstitium of the lymph nodes (Bogoslowski et al., 2020). They represent a phenotypically distinct subset of neutrophils when compared with circulating neutrophils with a high level of major histocompatibility complex II (MHCII)^*high*^ with the potential of influencing the adaptive immune system (Lok et al., 2019). IVM revealed that under homeostatic conditions a small population of neutrophils (∼1000 neutrophils per lymph node) continuously enter the lymph nodes via the high endothelial venules (HEV) in an L-selectin-dependent manner and leave the organ via efferent lymphatic in a sphingosine-1-phosphate (S1P)-dependent way (Bogoslowski et al., 2020). In contrast to other organs, neutrophil entry into the lymph nodes did not follow circadian rhythm. These temporarily resident neutrophils survey the tissue for pathogens and following bacterial infection, recruit additional neutrophils but not after sterile injury suggesting that lymph node neutrophils are able to discriminate the nature of the insult and respond accordingly (Bogoslowski et al., 2020).

Intravital microscopy has been pivotal in documenting the dynamic influx of neutrophil from inflamed tissues into the lymph nodes in response to infection (Hampton et al., 2015; Bogoslowski et al., 2020). Circulating and tissue-resident neutrophils have been shown to use both the afferent lymphatics of the infected tissue as well as HEV to enter the lymph nodes (Chtanova et al., 2008; Duffy et al., 2012; Gorlino et al., 2014; Hampton et al., 2015; Bogoslowski et al., 2020). The neutrophils that are able to migrate to lymph nodes and to modulate adaptive immune reactions express CD11b^*high*^, CD62L^*low*^, and CXCR2^*low*^. They seem to use different molecular mechanism to enter the lymph nodes; CCR7 is essential for neutrophils to enter via afferent lymphatics (Beauvillain et al., 2011), while L-selectin is essential for neutrophil entry via HEV (Gorlino et al., 2014; Bogoslowski et al., 2020). The molecular mechanisms of neutrophil entry and their physiological and pathological implications have been reviewed by Voisin and Nourshargh (2019).

Intravital microscopy showed the entry of neutrophils into the popliteal lymph node (PLN) via multiple hotspots on HEV following influenza vaccination (Pizzagalli et al., 2019). Moreover, neutrophil positive for influenza virus were tracked entering into the PLN after vaccination and showed over a time of 2 h changes in their dynamic motility with a decrease in instantaneous and mean speed, directionality, displacement, and an increase in the arrest coefficient suggesting an increase in cell-to-cell interactions (Pizzagalli et al., 2019). Five distinct neutrophil migratory behaviors have been observed: flowing, arrested, patrolling, directed migration, and swarming (Pizzagalli et al., 2019). During swarming, neutrophils were observed forming clusters that enlarged over time in areas rich in tissue resident macrophages (Pizzagalli et al., 2019). In another study using a model of skin infection, neutrophils were observed migrating to the PLN recruited by tissue resident macrophages in an IL-1β-dependent manner to control the spread of pathogens (Kastenmuller et al., 2012).

In a model of *S. aureus* infection, imaging of the inguinal lymph nodes by IVM revealed a dynamic influx of neutrophils that occurred in two waves with the second one composed of neutrophils mobilized by the BM (Kamenyeva et al., 2015). Moreover, by imaging lymphocytes, neutrophils and fluorescently labeled pathogens at the same time, Kamenyeva et al. (2015) showed that neutrophils interact extensively and directly with lymph node resident B cells to dampen their IgG and IgM production.

Applying IVM has also revealed the remarkably coordinated movement of two consecutive neutrophils, called “two-neutrophil squads” within the small capillaries of lymph nodes and found that these innate immune cells use alternative branches at bifurcations in order to avoid the formation of “traffic jam” within the same branch (Wang et al., 2020). Moreover, when four consecutive neutrophils enter a capillary with branches, two alternative patterns were observed, left-right-left-right or vice versa. This pattern has been explained by the fact that when a neutrophil is traveling along a capillary of the lymph node, it reduces the chemoattractant gradient in the capillary segment where it has just traveled in and increases the hydraulic resistance of the capillary it is occupying, hence the following neutrophil uses the opposite branch to continue its journey where the chemokine gradient is higher and hydraulic resistance lower (Wang et al., 2020).

Two-photon scanning-laser microscopy has provided information on the coordinated migration pattern of neutrophils within the draining lymph nodes after tissue infection showing that neutrophils can swarm and form small, large, transient, or persistent clusters within the lymph nodes (Chtanova et al., 2008). Visual imaging over time revealed that neutrophils show a direct migration rather than random within the lymph node with a high average speed of 11.9 μm/min to form clusters even from a distance of more than 70 μm from the swarm center (Chtanova et al., 2008). Moreover, visual imaging helped in defining that neutrophil swarming is initiated by pioneering neutrophils that come together within the first minutes followed by a massive influx of neutrophils later on (Chtanova et al., 2008; Lammermann, 2016). Neutrophil persistence within the lymph nodes disrupted the continuous layer of CD169^+^ macrophages present in the sub-capsular sinus suggesting tissue remodeling by infiltrated neutrophils (Chtanova et al., 2008). Making use of a photoconvertible system, Kikume reporter mouse, and two-photon microscopy, the fate of neutrophils first recruited to the inflamed skin and second into the lymph nodes have been imaged and showed a crawling speed of ∼13 μm/min via a CD11b and CXCR4-dependent mechanism (Hampton et al., 2015).

These data show that not all the neutrophils that infiltrate infected areas die *in situ*. At least some can re-enter either the blood vessels or the lymphatics and localize within the draining lymph nodes. These exciting studies show that neutrophils have many more functions beyond the direct killing of pathogens and tissue repair, including the recruitment and activation of other leukocytes, modulation of the adaptive immune system, antigen presentation, and blocking pathogen dissemination beyond the lymph nodes.

## Senescence and Neutrophil Death

In the aging process of a cell, senescence represents a step before apoptosis. Senescent neutrophils characterized by an increase in cell surface level of CXCR4 selectively return to the BM at the end of their life for clearance (Martin et al., 2003; Furze and Rankin, 2008b). Moreover, circulating senescent neutrophils are characterized by Ly6G^+^ CD62L^*low*^. Applying IVM to the calvarium to follow these aged neutrophils within the BM revealed these cells have a high migratory capability, supported by the upregulation of CD11b^*high*^/CD49d^*high*^. Moreover, 40% of Ly6G^+^ CD62L^*low*^ was found in direct contact with CD169^+^ BM macrophages for their final clearance but far away from CAR cells (Evrard et al., 2018). This mechanism of clearance represents a homeostatic signal that modulates hematopoietic niches in the BM and that regulates appearance of progenitors into the circulation (Casanova-Acebes et al., 2013; Adrover et al., 2016). Moreover, the spatial difference in location of “fresh” and aged neutrophils supports the idea that the process of neutrophil maturation and clearance of senescent neutrophils happens in spatially separated areas of the BM, with special areas for maturation and retention of freshly produced neutrophils and others for phagocytosis of aged neutrophils.

Under homeostasis, spontaneous clearance of CD62L^*low*^ neutrophils from the circulation follows a circadian rhythm with an accumulation during the light time between zeitgeber times (ZT) ZT5-ZT13 and clearance from the circulation during the night time ZT17-ZT5 in mice (Casanova-Acebes et al., 2013). While many studies have successfully imaged the BM via IVM, capturing the migration of neutrophils across the BM sinusoidal endothelium as they are mobilized into the blood, the uptake of senescent neutrophils by macrophages has proven extremely challenging, as well as quantitative analysis of these processes (Adrover et al., 2016).

Interestingly, the molecular profile of senescent neutrophils CD62L^*low*^ and CD11b^*high*^ resembles one of the activated neutrophils. As suggested by Casanova-Acebes et al. (2013), aged neutrophils could share a common program of activation signature to ensure the return of neutrophils to the BM for clearance. In fact, there are several studies showing that compromised clearance of cells leads to an unbalanced homeostasis and loss in vascular protection (Adrover et al., 2019). Thus in a model of zymosan-induced peritonitis, aged neutrophils showed an impaired ability to migrate within inflamed tissues, while retaining an ability to migrate toward tissues that support clearance suggesting a selective recruitment of “fresh” neutrophils for fighting infections (Adrover et al., 2019).

Neutrophil-specific deletion of CXCR2 or CXCR4 shows a disruption in the temporal changes of CD62L level on neutrophils. Circulating neutrophils of mice CXCR2^Δ*N*^ express constitutively high level of CD62L, while in CXCR4^Δ*N*^ constitutively low CD62L. Further CXCR2 has been shown to promote aging during the day while CXCR4 prevents it, suggesting that both chemokine receptors are responsible for controlling the process of neutrophil aging (Adrover et al., 2019).

Bone marrow is not the only organ where aged neutrophils can be cleared. This function is equally shared between the BM, spleen and liver (Suratt et al., 2001; Furze and Rankin, 2008b). However, the molecular mechanism of senescent neutrophil clearance in the spleen and liver is not fully understood (Furze and Rankin, 2008b). Aged neutrophils are cleared in the liver by tissue resident macrophages (Kupffer cells) in a G-α_*i*_-independent manner, while in the spleen half of senescent neutrophils are cleared in a G-α_*i*_-independent manner and half in a G-α_*i*_-dependent manner; however, the receptor involved is still not known (Furze and Rankin, 2008b). IVM imaging of the spleen treated with AMD3100 showed an increased number of splenic neutrophils but not activation or changes in CXCR4 expression suggesting that the process of senescent neutrophil clearance in the spleen is CXCR4 independent (Pillay et al., 2020).

## Development of New Therapeutics

**FIGURE 3:**
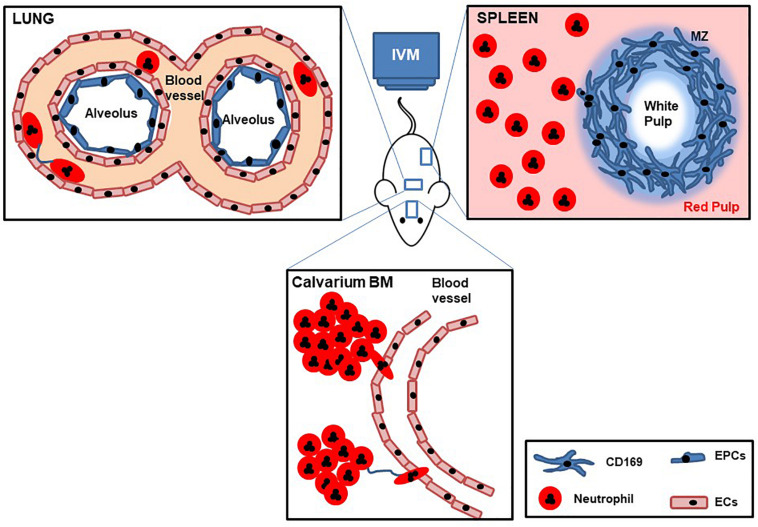
Cartoon with schematic representation of lung, spleen, and calvarium BM IVM. Mice are deeply anesthetized, fluorescent Abs are intravenously injected, and internal organs are partially exposed to be imaged by IVM while maintaining blood flow. Dynamic behaviors of tissue neutrophil motility, cell–cell interactions, and temporal change in speed are recorded over time.

The dynamics and molecular mechanisms underlying neutrophil trafficking in homeostasis and disease have been extensively studied by IVM over the last decades, with technological advances allowing researchers to continually uncover new functions and facets of these fascinating cells (Figure 3). Thus while the original seminal studies led to the generation of the adhesion cascade paradigm (tethering rolling, adhesion transmigration) increasingly more sophisticated IVM, together with availability of fluorescent reporter mice, and genetically modified mice has revealed increasing levels of complexity to this process (Girbl et al., 2018). Moreover, technological advances that have allowed imaging of tissues including the lung, spleen, and lymph nodes have led to an understanding that neutrophil responses are both tissue and pathogen specific, but moreover that there are distinct subsets of neutrophils in these tissues that have different responses and functions. Finally, the temporal nature of neutrophil responses has been revealed by IVM, whether that is early versus late response to a pathogen, or differential responses dependent on the time of day or night. This ever increasing level of complexity means that we are now in a stronger position to understand neutrophil related diseases and design targeted therapies. Going forward IVM is a technique that could help to test *in vivo* the mechanism of action of drugs and help design more potent or specific therapeutics, as has been shown recently with the identification of DEPEP1 the adhesion molecule for neutrophil retention in the lungs following LPS challenge, that could be a potential therapeutic target for ARDS (Choudhury et al., 2019). In addition to having more knowledge to specifically ameliorate inflammatory pathologies we can now start to understand in more detail, the function of tissue resident neutrophils, the different subsets of these cells, and the choreography of their recruitment, tissue retention, and maturation thanks to IVM.

## Author Contributions

KD performed the literature search and wrote the manuscript. SR contributed to the writing and revision of the manuscript. Both authors contributed to the article and approved the submitted version.

## Conflict of Interest

The authors declare that the research was conducted in the absence of any commercial or financial relationships that could be construed as a potential conflict of interest.
